# Neuroinflammatory and transcriptional dynamics during SARS-CoV-2 infection in KRT18-hACE2 mouse brain

**DOI:** 10.3389/fimmu.2026.1716597

**Published:** 2026-02-02

**Authors:** Dae-Gyun Ahn, Nhu Thi Quynh Mai, Da-Jin Jeong, Byoung-San Moon

**Affiliations:** 1 Department of Convergent Research of Emerging Virus Infection, Therapeutics and Biotechnology Division, Korea Research Institute of Chemical Technology, Daejeon, Republic of Korea; 2 Department of Medical Biotechnology, Yeungnam University, Gyeongsan, Republic of Korea

**Keywords:** chemokines, cytokines, immune signaling, inflammation, neuroinvasion, SARS-CoV-2

## Abstract

**Introduction:**

Neurological complications are increasingly recognized as a significant consequence of COVID-19; however, time-resolved, brain-specific characterization of transcriptional alterations underlying SARS-CoV-2–associated neuroinflammation and neuronal injury remain limited. We hypothesized that brain transcriptional responses evolve dynamically during acute SARS-CoV-2 infection, resulting in temporal transcriptional programs.

**Methods:**

KRT18-hACE2 transgenic mice were intranasally inoculated with SARS-CoV-2. Brain was harvested at 4 and 6 days post-infection (dpi) for analyses.

**Results:**

Immunohistochemical analyses confirmed a broad spectrum of viral neurotropism and gliotropism, accompanied by an increased apoptotic burden, particularly in cortical neurons (ClCas3/SATB2+). Robust activation of myeloid cells (Iba1+/CD68+) provided evidence of neuroinflammation. Cytokine/chemokine profiling demonstrated pronounced upregulation of inflammatory mediators (CXCL10, IL-12p40, CCL12), alongside reduced CX3CL1, suggesting impaired neuron–microglia communication. Whole-transcriptome and gene ontology analyses uncovered stage-dependent molecular programs, with early alterations at 4 dpi enriched in protein ubiquitination, vesicle trafficking, and synaptic processes, followed by intensified innate immune activation and engagement of chromosomal maintenance pathways at 6 dpi. In parallel, pronounced suppression of mitochondrial function at 6 dpi, pointing to energy exhaustion and transcriptional-translational discordance, as supported by digital PCR and a substantial reduction in COXIV protein levels.

**Discussion:**

These findings provide a time-resolved molecular landscape of SARS-CoV-2–induced neuroinflammation and metabolic stress, highlighting CNS vulnerability during severe infection and suggesting pathways potentially relevant to COVID-19-associated sequelae.

## 1 Introduction

COVID-19, caused by SARS-CoV-2, has become a global health crisis, placing unprecedented strain on healthcare systems and economies worldwide (1, 2). Although primarily a respiratory disease, accumulating evidence indicates that SARS-CoV-2 can affect central nervous system (CNS) (3, 4), where it is capable of triggering neuroinflammation (5, 6) and contributing to persistent neurological and psychiatric sequelae (7). Clinical manifestations span a broad spectrum, ranging from anosmia and ageusia to severe neurological conditions such as encephalitis, seizures, and cerebrovascular events (8, 9). These understandings mark SARS-CoV-2 as a significant, though relatively indirect, neurotropic virus, underscored by its capacity to perturb CNS homeostasis. Importantly, converging clinical, pathological, and experimental data suggest that direct viral neurotropism is not the principal driver of most COVID-19-associated neurological sequelae; rather, indirect mechanisms, including cytokine dysregulation, endothelial and blood-brain barrier disruption, microthrombosis, and hypoxia, appear to play dominant pathogenic roles (10–13), with direct CNS infection contributing in a subset of cases, potentially acting as a triggering factor (14). Despite this growing body of evidence, the molecular and transcriptional programs underlying virus-driven neuroinflammation remain incompletely characterized, particularly in a time-resolved manner.

hACE2 transgenic mouse models, which permit SARS-CoV-2 entry via the ACE2 receptor, are widely used to investigate CNS pathogenesis and have provided evidence for viral neuroinvasion, neuroinflammation, and neuronal injury (15–19). SARS-CoV-2 infection progresses through distinct phases, each characterized by dominant viral and host processes, including induction of inflammatory gene programs (19), elevated cytokine and chemokine expression in the brain (17), and progressive manipulation of host cellular machinery (20, 21). Notably, epidemiological and biomarker studies have indicated that SARS−CoV−2 infection is associated with an increased risk of subsequent neurodegenerative diagnoses and with sustained markers of neuronal and astroglial injury in long−COVID cohorts (22, 23), suggesting a potential link between neuro-COVID and neurodegenerative-like brain changes that extends beyond viral burden alone, although causality and the precise mechanistic cascade remain to be fully elucidated. Temporal interrogation of inflammatory and transcriptional dynamics is essential for understanding the evolution of SARS-CoV-2 associated neuropathology. In this context, previous comparative analyses on beta over original variant has reported time-dependent alterations in systemic and CNS immune responses (24). However, a time-resolved characterization of brain-specific transcriptional programs at key disease stages remains limited, and such insights are critical for understanding SARS-CoV-2 neuropathogenesis and for identifying therapeutic targets aimed at mitigating both acute and long-term neurological complications.

Here, we aimed to characterize the neuropathological and transcriptional responses in the brains of KRT18-hACE2 transgenic mice following intranasal SARS-CoV-2 infection, focusing on two critical stages, 4- and 6-days post-infection (dpi), which correspond to peak viral replication and heightened immune activation preceding lethal diseases in this model. By integrating cytokine/chemokine profiling with whole-transcriptome profiling, gene ontology analysis and absolute quantification of selected targets by digital PCR, we delineate time-dependent neuroinflammatory and metabolic remodeling. Our analyses reveal stage-specific transcriptional programs and RNA–protein discordance in key inflammatory and mitochondrial pathways, highlighting complex regulatory mechanisms underlying SARS-CoV-2–induced neuroinflammation. Collectively, these findings provide a descriptive, time-resolved molecular landscape that informs mechanisms of acute neuro-COVID and lays the groundwork for the development of stage-specific therapeutic strategies aimed at mitigating both acute and long-term neurological complications.

## 2 Materials and methods

### 2.1 Animals and viral infection

All work involving live SARS-CoV-2 was conducted in a Biosafety Level 3 (BSL-3) containment facility, in accordance with approved safety protocols and with the use of appropriate personal protective equipment. Animal procedures were approved by the Institutional Animal Care and Use Committee (IACUC) of the Korea Research Institute of Chemical Technology (permit no. 2021-8A-04-01, protocol no. 8A-M6). Male K18-hACE2 mice (no. 034860) [B6.Cg-Tg(K18-ACE2)2Prlmn/J] were purchased from the Jackson Laboratory. Mice were housed in groups under standard laboratory conditions with free access to food and water and were randomly divided into groups before infection.

SARS-CoV-2 wild-type strain (BetaCoV/Korea/KCDC03/2020) (GISAID accession number EPI_ISL_407193) was provided by the National Culture Collection for Pathogens (25) (NCCP; numbers 43326 and 43382) of the Korea Disease Control and Prevention Agency. For SARS-CoV-2 infection, mice were anesthetized with isoflurane and injected intranasally with 50 μL of saline containing 2 × 10^3^ PFU of SARS-CoV-2 wild-type strain (25 μL per nostril). Control mice were mock-infected with vehicles. This inoculum represents a sublethal respiratory dose but is the minimum required to induce reproducible and consistent neuroinvasion and CNS pathology in K18-hACE2 mice. Weight loss, individual clinical scores and mortality were monitored daily after infection and reported in our previous study (24).

### 2.2 Tissue collection

Mice were sacrificed at 4- and 6- day post-infection (dpi). Uninfected and SARS-CoV-2-infected mice were anesthetized with isoflurane. Whole brain was perfused with 10 mL of phosphate-buffered saline (PBS) to remove blood. For immunofluorescence, brains were fixed in 10% neutral buffered formalin at room temperature for 2 to 5 days. For microarray and cytokine/chemokine arrays, brains were homogenized in cold PBS using a FastPrep-24 homogenizer (MP Biomedicals) for three cycles of 15 s on and 20 s off. Tissue debris was removed by brief centrifugation.

### 2.3 Immunohistochemistry

Fixed brain samples were embedded in paraffin blocks, and 5-μm-thick sagittal sections of hemisphere were generated using a rotary microtome (Roundfin RD-315, Shenyang, China), mounted on glass slides, and allowed to dry. Prior to staining, sections were deparaffinized and rehydrated by sequential immersion in three changes of xylene, followed by xylene:100% ethanol (1:1, v/v), 100%, 95%, and 80% ethanol (10 min each), and finally distilled water. Antigen retrieval was performed by boiling the sections in citrate buffer (10 mM sodium citrate, 0.05% Tween-20, pH 6.0) for 20 min. After cooling to room temperature, samples were blocked with 5% bovine serum albumin (BSA) for 30 min and incubated with primary antibodies overnight at 4°C. The following day, sections were washed three times with PBS (10 min each), incubated with fluorescently labeled secondary antibodies diluted in 1% BSA for 1 h at RT, and counterstained with 4′,6-diamidino-2-phenylindole (DAPI, 100ng/mL). Finally, slides were washed twice (10 min each), mounted, and imaged using K1 Fluo confocal microscopy (Nanoscope Systems, Inc., Korea). Images were acquired with a 40× objective lens and a resolution of 2048 × 2048 pixels. For each marker, acquisition parameters were optimized using control sections and kept constant for 6dpi group.

Three mice per group (n = 3) were analyzed, with three brain sections per mouse examined, focusing on cortical regions. Quantification was performed manually in a blinded manner across a defined number of high-power fields per section. Marker-positive and double-positive cells were identified based on fluorescence intensity above background and cellular morphology, and results were expressed as the percentage of positive cells relative to DAPI-positive nuclei. Representative images were selected to reflect the quantified results.

Primary antibodies used in this study included anti-NP (Cell Signaling Technology, 33717), anti-SATB2 (Abcam, ab34735), anti-MAP2 (Abcam, ab183830), anti-GFAP (Abcam, ab7260), anti-O4 (R&D system, MAB1326), anti-Iba1 (Cell Signaling Technology, 58970), anti-CD68 (Cell Signaling Technology, 97778), anti-Cleaved Caspase 3 (Cell Signaling Technology, 9661), all used at a dilution ratio of 1:500.

Secondary antibodies used in this study included goat anti-rabbit Alexa Fluor 488-conjugated IgG (Abcam, ab1500770), goat anti-rabbit Alexa Fluor 555-conjugated IgG (Abcam, ab1500780), goat anti-mouse Alexa Fluor 488-conjugated IgG (Abcam, ab150113), goat anti-mouse Alexa Fluor 555-conjugated IgG (Abcam, ab150114), all used at a dilution of 1:300.

### 2.4 TUNEL assay

DNA fragmentation was detected on rehydrated paraffin-embedded sections using a one-step TUNEL *in situ* apoptosis detection kit (E-CK-A325; Elabscience), according to the manufacturer’s instructions. The TUNEL assay was performed on the same set of tissue sections used for immunohistochemical analyses.

### 2.5 Proteome profile analysis

Five mice per group (n=5) were analyzed. Supernatant of brain homogenates were treated with 1% Triton X-100 to inactivate SARS-CoV-2 (15). Initial screening of 111 inflammatory proteins was performed using Proteome Profiler Mouse XL Cytokine Array (ARY028, R&D System), according to the manufacturer’s instructions. Chemiluminescent signals were detected and quantified using Quick Spots Tool (Western Vision software, Version 22.0.1b) and presented as relative fold-change in signal intensity compared with uninfected control.

### 2.6 Total RNA extraction & cDNA synthesis

The control group consisted of five mice (n=5), the S4d and S6d groups each comprised six mice (n=6). Total RNA was isolated from homogenized tissues using Maxwell RSC SimplyRNA Tissue Kit (Promega) according to the manufacturer’s instructions. RNA concentration and purity were assessed using a NanoDrop spectrophotometer (Nano400A; Allsheng). Samples that passed quality control (A260/A280 > 1.6) were used for cDNA synthesis using iScript Reverse Transcription Supermix for RT–qPCR (Bio-Rad) and a Mastercycler (Eppendorf). The resulting cDNA samples were used for whole-transcriptome analysis and digital PCR.

### 2.7 Whole-transcriptome analysis

Whole-transcriptome analysis was performed as in prior study using the Affymetrix GeneChip Mouse Gene 2.0 ST array (no. 902463) and GeneChip WT Pico kit (no. 902623) (Thermo Fisher Scientific) according to the manufacturer’s instructions. The raw data were summarized and normalized using the Robust Multi-Average method in Affymetrix Power Tools, followed by differential expression analysis. Statistical significance was determined using an independent *t* test and fold change. The null hypothesis was that no difference exists between the groups. The false-discovery rate was controlled by adjusting the *P* value using the Benjamini-Hochberg algorithm.

### 2.8 Differential expression analysis

We determined the statistical significance of the DEGs by performing pairwise contrasts between the 4dpi or 6dpi groups compared to the uninfected group. Genes with p-adj <0.05 (t-test) and ∣log2​FC| >1 were defined as differentially expressed genes. The resulting upregulated and downregulated gene lists for each experimental comparison were then used for Gene Ontology (GO) enrichment analysis via the ShinyGo web tool (http://geneontology.org). All data analyses and visualization were performed in R 3.3.2 (www.r-project.org).

### 2.9 Digital PCR

Absolute quantification of copy number of each target gene in each sample was performed using Absolute Q™ DNA Digital PCR Master Mix (ABI, Thermo Fisher Scientific) and QuantStudio Absolute Q Digital PCR System (ABI) following manufacture’s procedure. The primers used for dPCR were provided in **Supplementary Table 1**.

### 2.10 Statistical analysis

All the statistical analyses were performed via GraphPad Prism 9 software. The data were tested for normal distribution and within-group variance prior to statistical analysis. The data obtained from at least three independent experiments were averaged and are presented as the means ± SDs as stated in the figure legends. In all cases, a p value less than 0.05 was considered significant: *P < 0.05, **P < 0.01, ***P < 0.001.

## 3 Results

### 3.1 Neuroinvasion and cellular tropism of SARS-CoV-2 in the brain of KRT18-hACE2 transgenic mice

**Figure 1:**
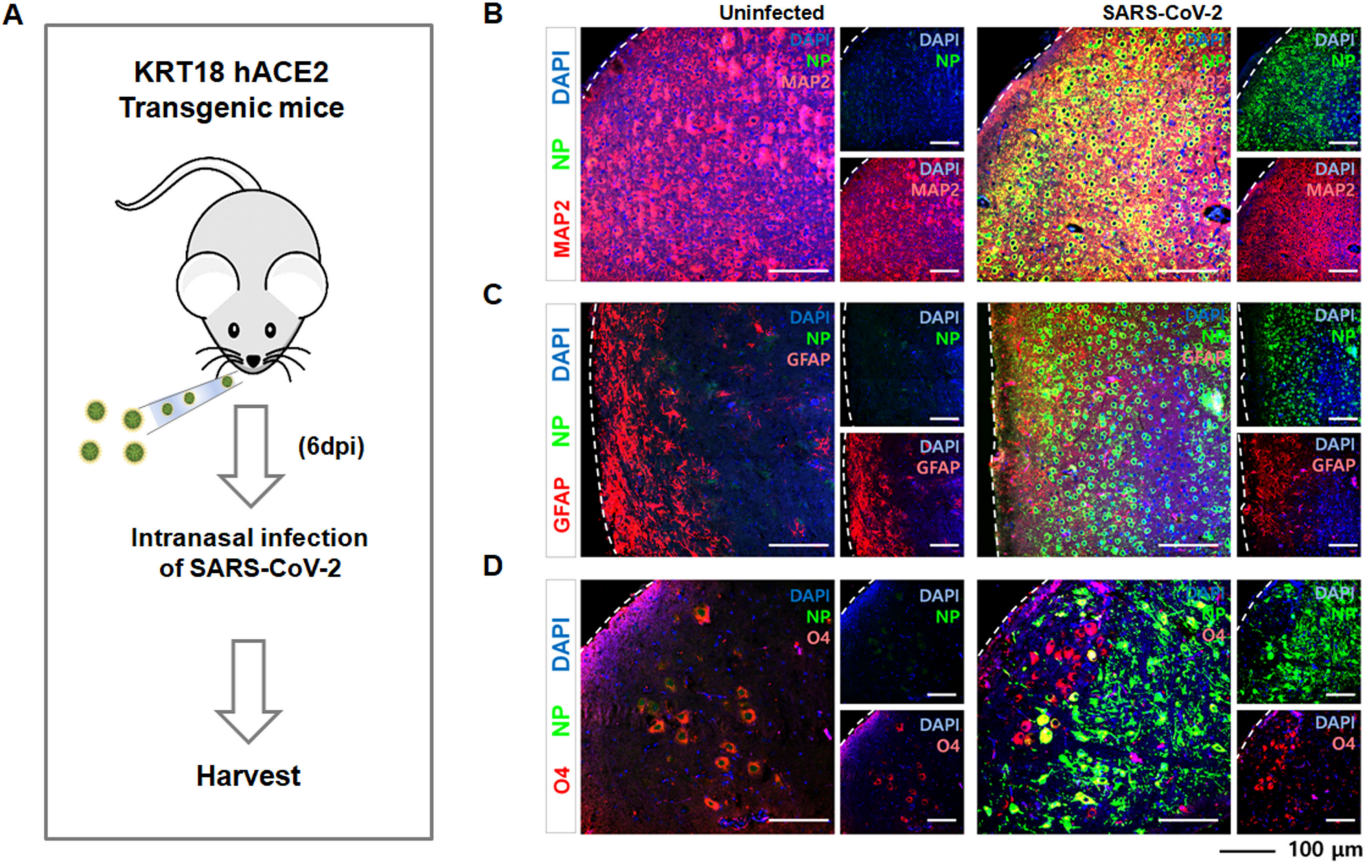
Neuroinvasion and cellular tropism of SARS-CoV-2 in a mouse model. **(A)** Schematic illustration of experimental design. KRT18-hACE2 transgenic mice were intranasally infected with SARS-CoV-2 and brain tissue was harvested six days post-infection (6 dpi). **(B-D)** Immunofluorescence staining of brain tissue from uninfected and infected KRT18-hACE2 transgenic mice. Each panel shows co-localization of DAPI (blue, nuclear stain) with SARS-CoV-2 nucleocapsid protein (NP, green) and specific **(B)** mature neuron MAP2 marker (red), **(C)** astrocyte GFAP marker (red), **(D)** oligodendrocyte O4 marker (red).

Previous studies, including our own, have shown that K18-hACE2 mice develop severe clinical disease and mortality around 5–6 days after SARS-CoV-2 infection, with peak viral burden and widespread brain distribution occurring at 6–7 days post-infection (dpi) (26, 27). Therefore, 6dpi was chosen to capture pronounce inflammation and severe pathology status. Accordingly, to specifically interrogate the neuroinvasive phase, we first confirmed SARS-CoV-2 neuroinvasion in the brains of intranasally infected K18-hACE2 mice at 6 dpi (**Figure 1A**). IHC analysis revealed co-expression of the viral nucleocapsid protein (NP) with MAP2 (**Figure 1B**) and SATB2 (**Supplementary Figure S1A**), indicating infection of mature neurons and cortical neurons, respectively. In addition, NP+ GFAP+ astrocytes (**Figure 1C**) and NP+ O4+ oligodendrocytes (**Figure 1D**) were detected, demonstrating viral infection of multiple glial populations.

Together, these results confirm direct neuroinvasion by SARS-CoV-2 at a defined acute disease stage and reveal broad cellular tropism across neuronal and glial lineages, providing a cellular framework for subsequent analyses of transcriptional and functional disruption in the infected brain.

### 3.2 SARS-CoV-2–induced neuropathologic alterations in the brain of KRT18-hACE2 transgenic mice

**Figure 2:**
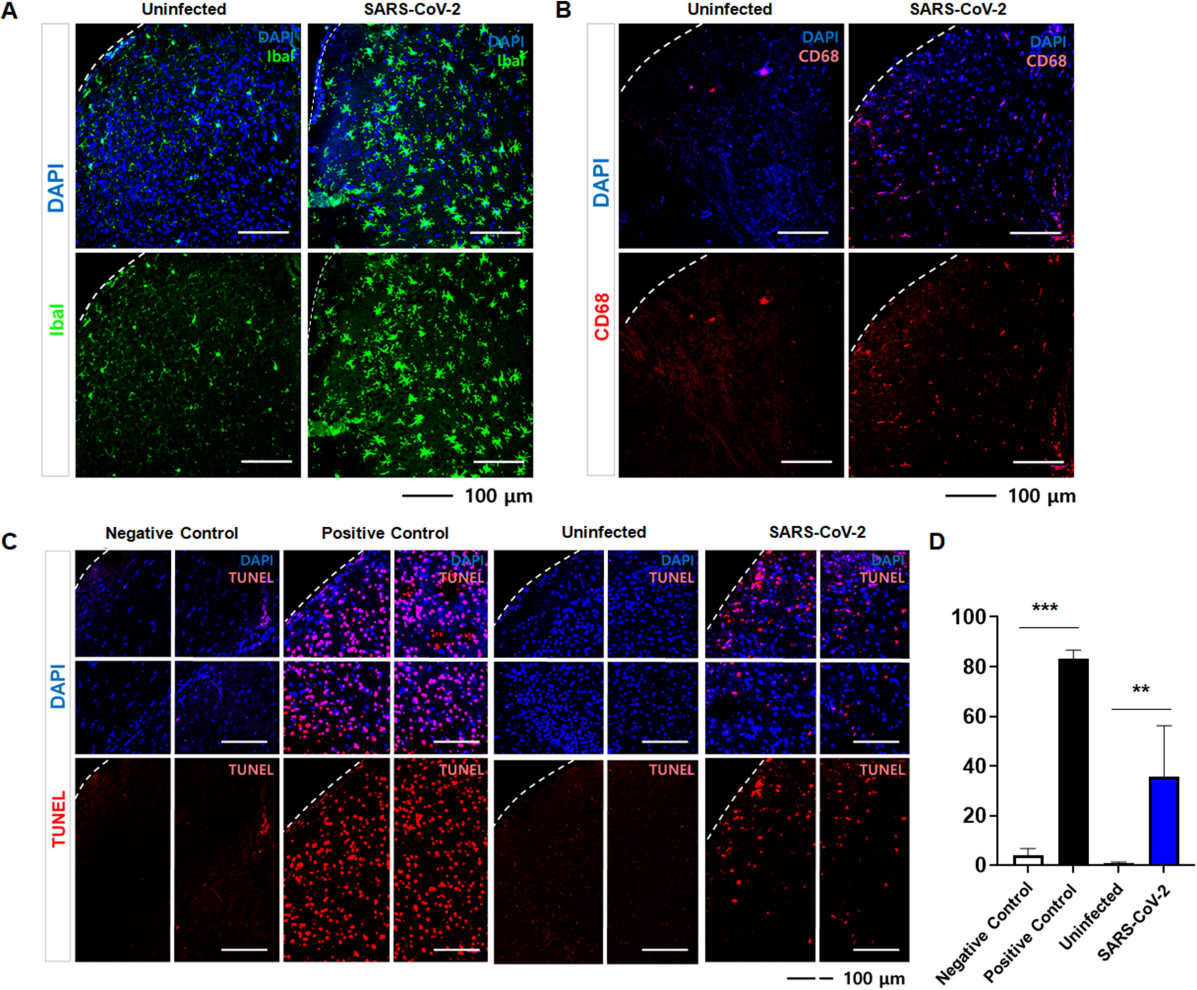
Neuropathological changes and inflammatory mediators in K18-hACE mouse brain following SARS-CoV-2 infection. **(A, B)** Immunofluorescence staining of brain tissue from uninfected and SARS-CoV-2 infected mice showing colocalization of DAPI (blue, nuclear stain) with **(A)** Iba-1 (green) and **(B)** CD68 (red). Lower panels show single staining for Iba-1 or CD68. Scale bar: 50 µm. **(C)** TUNEL assay of brain tissue from uninfected and SARS-CoV-2 infected mice. **(D)** Quantification of panel C showing the percentage of TUNEL+ cells in each group. Student t-test was performed to determine the significance. **p < 0.01, ***p < 0.001).

The identified neuro- and glio-tropic effects of SARS-CoV-2 prompted us to investigate neurodegenerative changes in the brain of KRT18-hACE2 mice following infection. To this end, we first assessed neuroinflammation as an early histopathological indicator. IHC analysis of 6 dpi brain revealed a marked increase in Iba1-positive cells (**Figure 2A**) and elevated CD68 expression (**Figure 2B**) compared with control, indicating activation of brain-associated myeloid cells, comprising both CNS-resident and infiltrating immune populations, and enhanced phagocytic activity. Consistently, infected brains exhibited an increased proportion of apoptotic cells, as demonstrated by TUNEL staining (**Figures 2C, D**), accompanied by a substantial increase in cortical neuronal apoptosis, evidenced by ClCas3/SATB2 double-positive cells (**Supplementary Figures S1B, C**).

Collectively, these results demonstrate that SARS-CoV-2 elicits robust immune responses in the brain and is associated with neurodegeneration, especially upper-layer cortical neurons, underscoring the contribution of inflammatory and apoptotic pathways to the neuropathological consequences of infection.

### 3.3 Enhanced immune response in SARS-CoV-2 infected brain of KRT18-hACE2 transgenic mice

**Figure 3:**
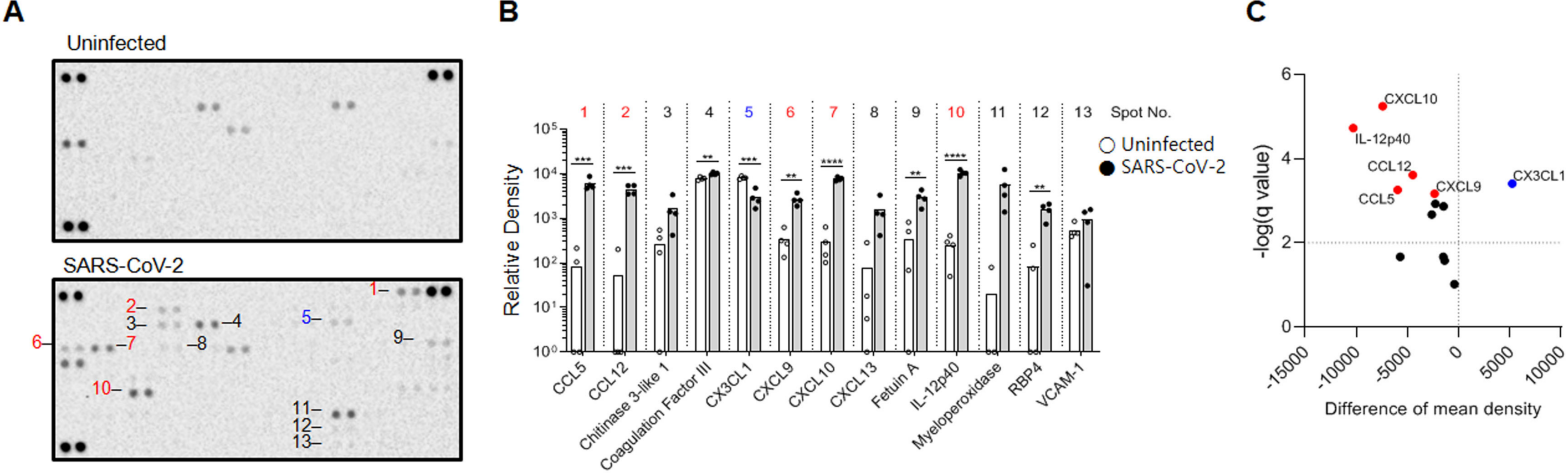
Cytokine and chemokine expression changes in SARS-CoV-2 infected brains. **(A)** Cytokine/chemokine antibody array showing protein expression in the brains of uninfected and SARS-CoV-2 infected mice. Each numbered spot corresponds to a specific cytokine or chemokine. **(B)** Quantification of the relative density of the spots in **(A)** Bars represent the relative density of each spot, indicating the expression level of the corresponding cytokine or chemokine. One-way ANOVA was performed to calculate significance. *p < 0.05, **p < 0.01, ***p < 0.001, ****p < 0.0001). **(C)** Volcano plot of differentially expressed cytokines/chemokines from **(B)** The axes represent the difference of mean density (fold change, x-axis) and statistical significance (-log10(q-value), y-axis). Labels for specific cytokines/chemokines of interest are shown.

To further characterize the inflammatory state of the brain at 6dpi, we performed proteomic profiling using a cytokine/chemokine targeting 111 analytes (**Figure 3A**). The results demonstrated a pronounce pro-inflammatory signatures, marked by the significant upregulation of the chemokines CCL5, CCL12, CXCL9, and CXCL10. In parallel, enhanced expressions of immune-associated proteins, including coagulation factor III, IL-12p40, and the acute-phase proteins Fetuin-A, were observed (**Figures 3B, C**). In contrast, expression of CX3CL1, a chemokine critical for maintaining the neuronal – microglial axis (28, 29), was reduced. This reciprocal pattern of increased pro-inflammatory mediators and decreased homeostatic signaling molecules suggests a shift toward an inflammatory state of the infected brain at 6 dpi. Furthermore, the elevated RBP4 expression, a key mediator of retinol signaling in the brain (30), plausibly pointing to metabolic disorders.

These data, therefore, demonstrate that SARS-CoV-2 infection is associated with an enhanced immune and inflammatory response in the brain at the protein level and suggest the association of metabolic signaling to neuropathological alterations.

### 3.4 Stage-dependent antiviral and immune transcriptional responses to SARS-CoV-2 infection in the brain of KRT18-hACE2 transgenic mice

**Figure 4:**
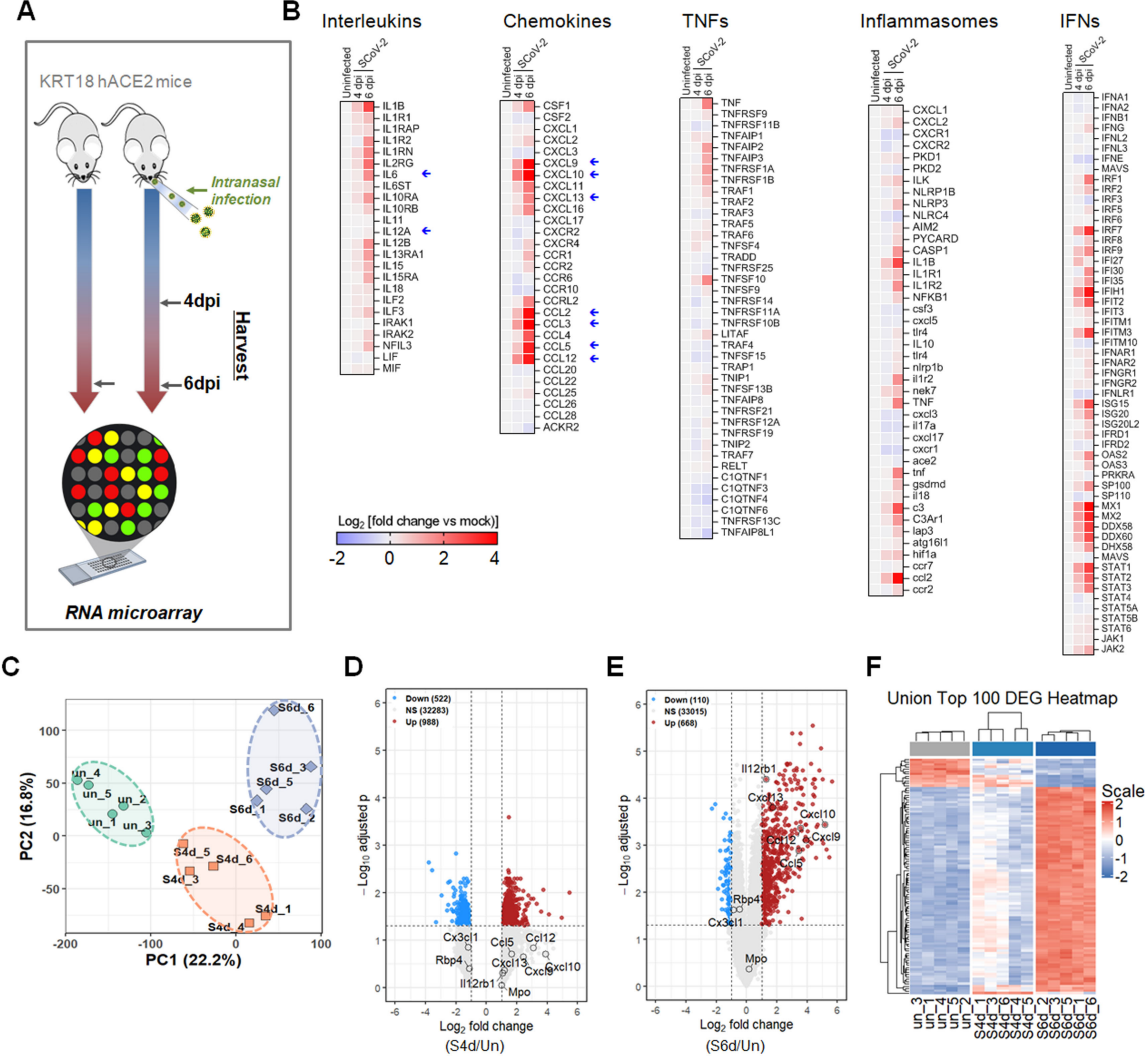
Stage-dependent antiviral responses to SARS-CoV-2 infection in the brain of KRT18-hACE2 transgenic mice. **(A)** Schematic overview of experimental design. **(B)** Heat map demonstrating RNA level of each gene in each experimental condition (Uninfected, 4dpi, 6dpi). **(C)** PCA plot showing distinct clustering patterns among RNA microarray profiles for each experimental group. **(D, E)** Volcano plot displaying the log2FC values of the differentially expressed genes in the **(D)** 4dpi and **(E)** 6dpi group versus the control group. **(F)** Heatmap displaying differential RNA expression profiles across Uninfected, 4 dpi and 6 dpi groups, highlighting distinct transcriptional changes over the course of SARS-CoV-2 infection.

Our prior studies (24) and published literatures (26, 27) demonstrated that 6 dpi represents fully developed acute disease phase with maximal inflammation and imminent mortality, whereas 4 dpi corresponds to peak viral replication and early inflammatory responses in K18-hACE2 mice, we asked whether transcriptomic alterations also occur in a time-resolved manner. To examine the temporal dynamics underlying the development of immune and inflammatory responses during infection, RNA microarray analyses were performed at 4 dpi, representing early immune activation, and at 6 dpi, capturing severe pathology and mortality (**Figure 4A**). Consistent with the proteomic profiling, most immune-related transcripts were more prominently upregulated at 6dpi, indicating a stage-dependent escalation of immune activation (**Figure 4B**). Specifically, transcripts associated with interleukins, chemokines, TNF signaling, inflammasomes, and interferon pathways were markedly elevated post-infection, particularly at 6 dpi, highlight the progressive immune activation in the brain. Principal component analysis (PCA) further supported this observation, showing distinct stage-specific clustering that separated uninfected from infected groups at both time points (**Figure 4C**). Volcano plots for diffentially expressed genes (DEGs) analysis relative to the uninfected group further demonstrated a substantially broader range of up- and downregulated transcriptional signatures in 6dpi compared to 4 dpi (**Figures 4D, E**). Notably, transcripts that were significantly altered at 6 dpi showed no significant changes at 4 dpi, indicating extensive dynamic remodeling of the host responses over the course of infection. Identification of the top 100 DEGs across three groups (**Figure 4F**) emphasized clear expression differences between 4dpi and 6dpi, further supporting a time-dependent pathological transition in the KRT18-hACE2 mouse brain following SARS-CoV-2 invasion.

### 3.5 Early-stage transcriptional remodeling in the brain at 4 dpi

**Figure 5:**
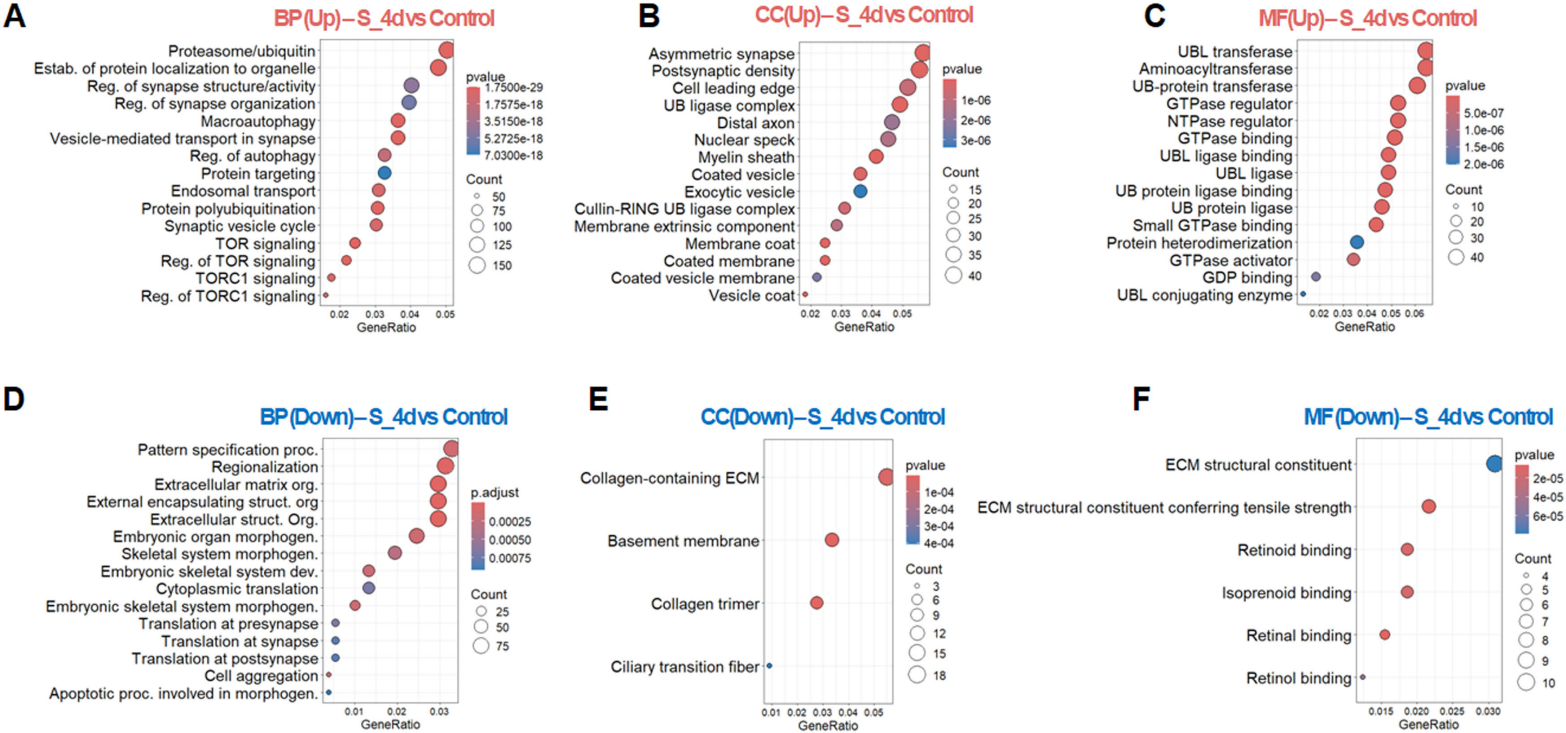
Comparative gene ontology analysis of brains at 4 dpi relative to uninfected controls. **(A-C)** Top 15 significantly upregulated terms in each category: **(A)** biological process (BP), **(B)** cellular component (CC), **(C)** molecular function (MF); **(D-F)** Top 15 significantly downregulated terms in each category: **(D)** BP, **(E)** CC, **(F)** MF. The results illustrate enriched pathways and molecular features associated with SARS-CoV-2-induced transcriptional alterations.

To further dissect the stage-dependent immune response to SARS-CoV-2 infection, we performed gene ontology (GO) enrichment analysis of DEGs at each stage. At 4dpi, upregulated biological processes (BP) terms were predominantly enriched for pathways related to protein biogenesis and turnover, as well as synaptic organization and plasticity (**Figure 5A**). MA plot analysis supported these enrichments, highlighting the top 10 marker genes within each selected pathway such as proteasome/ubiquitin, endosomal transport (**Supplementary Figure S2A**). Enrichment of macroautophagy-related terms was also observed. Upregulation was also notable in macroautophagy-related pathways, suggesting potential engagement of autophagy in response to viral infection and organelle stress. Additionally, GO terms related to synaptic vesicle cycling and TOR pathway were enriched among upregulated genes, indicating transcriptional changes affecting pathways involved in neuronal function at 4dpi. These transcription signatures are primarily associated with subcellular domains mediating ubiquitin-proteasome degradation, transcriptional regulation, particularly within neuronal compartments (**Figure 5B**), with prominent ubiquitination-related enzymatic activities (**Figure 5C**). Together, these findings indicate early transcriptional changes in pathways associated with protein homeostasis and vesicular processes in infected brain. Importantly, the high distribution within vesicular system together with promoted GTPase associated activities suggests the involvement of intracellular trafficking processes required for SARS-CoV-2 egress (31, 32), where GTPases play a critical role (33).

In contrast, downregulated GO terms at 4dpi were enriched for pathways governing cytoarchitecture, including neuronal patterning, regionalization and specialization (**Figure 5D**). The most strongly suppressed genes were associated with extracellular matrix (ECM) organization, cell aggregation, and morphogenetic programmed cell death (**Supplementary Figure S2B**). Downregulation of genes controlling local protein synthesis at presynapse sites was also detected, suggesting alterations in transcriptional program linked to synaptic plasticity. These downregulated transcripts were predominantly associated with ECM-related compartments (**Figure 5E**). Notably, GO terms related to retinoid pathways, a critical antiviral mechanism known to interfer with spike-mediated cell entry (34), were significantly suppressed at 4dpi (**Figure 5F**).

Collectively, these GO enrichment analyses reveal stage-specific transcriptional remodeling in the brain at 4dpi characterized by coordinated changes in gene sets associated with protein homeostasis, vesicular organization, and neuronal structural programs during the neurotropic phase of SARS-CoV-2 at 4 dpi.

### 3.6 Late-stage transcriptional remodeling in the brain at 6 dpi

**Figure 6:**
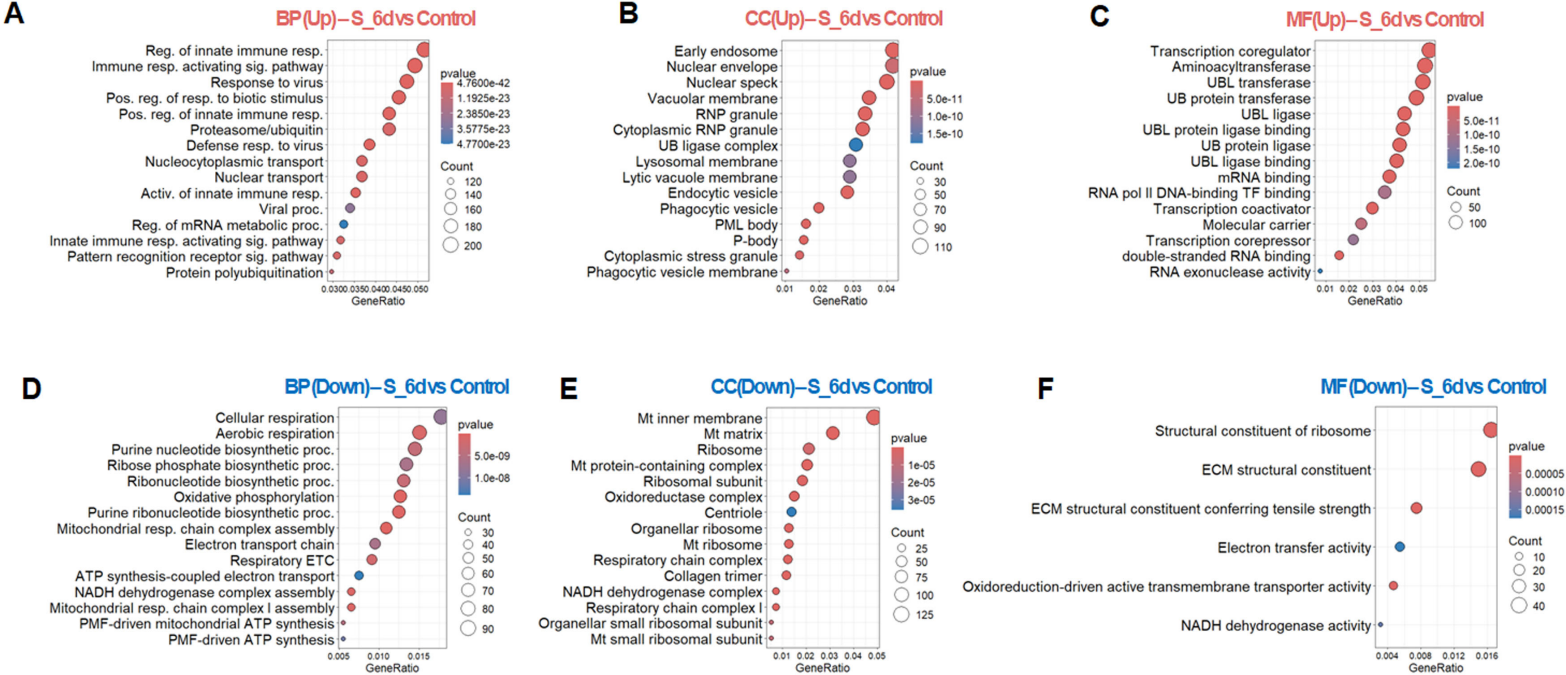
Comparative gene ontology analysis of brains at 6 dpi relative to uninfected controls. **(A-C)** Top 15 significantly upregulated terms in each category: **(A)** BP, **(B)** CC, **(C)** MF. **(D-F)** Top 15 significantly downregulated terms in each category: **(D)** BP, **(E)** CC, **(F)** MF. The results reflect enriched pathways and molecular features associated with SARS-CoV-2-induced transcriptional alterations.

At 6 dpi, transcriptomic signatures were predominantly enriched for innate immune processes in brain (**Figure 6A**; **Supplementary Figure S2C**). GO analysis revealed significant enrichment of genes sets associated with endolysosomal and phagocytic compartments, and activation of multiple RNA regulators localized to stress granules, P-bodies, and RNP granules (**Figure 6B**). These enrichments indicate broad transcriptional representation of pathways typically linked to antiviral and inflammatory responses.

In parallel, molecular function GO terms related to ubiquitin-like (UBL) ligases and transferases were significantly enriched (**Figure 6C**), reflecting transcriptional changes in pathways involved in protein modification and turnover. Additional enrichment was observed for pathways associated with nucleocytoplasmic and nuclear transport, and membrane-associated compartments, suggesting altered expression of genes involved in nuclear substructure such as PML bodies and nuclear specks, which are broadly associated with transcriptional regulation and nuclear organization.

Conversely, GO analysis revealed significant downregulation in multiple processes spanning different steps of cellular respiration at 6dpi (**Figure 6D**). Pathways involved in purine (ribo)nucleotide and ribose phosphate biosynthesis, tightly linked to ATP synthesis, were among the most suppressed. Consistent with these findings, downregulated genes were enriched in mitochondrial and mitochondria-associated cellular components (**Figure 6E**), including transcripts corresponding to multiple stages of mitochondrial respiration (**Supplementary Figure S1D**). Molecular function analysis further highlighted reduced representation of enzymatic activities essential for core metabolic processes, along with altered expression of genes associated with ECM organization (**Figure 6F**).

Together, these GO enrichment analyses indicate that late-stage SARS-CoV-2 infection is associated with coordinated transcriptional changes characterized by enrichment of immune-related pathways alongside suppression of metabolic and mitochondrial gene programs in the brain.

### 3.7 Time-resolved remodeling of immune- and mitochondria- associated genes

**Figure 7:**
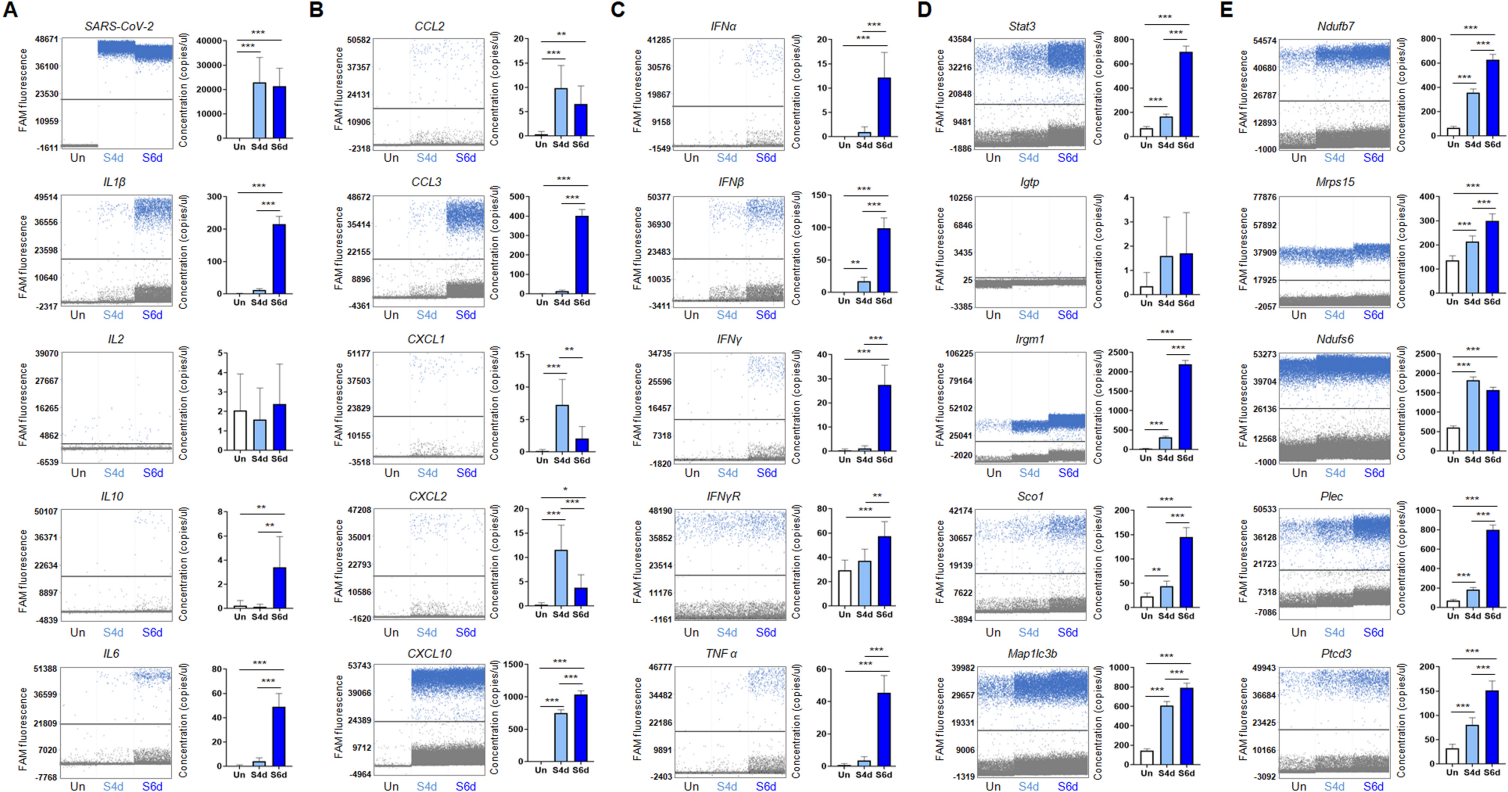
dPCR quantification of absolute copy numbers of selected genes in brain tissue at 4 and 6 dpi. The left panel displays representative fluorescence amplitude plots showing the distribution of individual droplets. The right panels present quantification of transcript copy concentrations (copies/µL) for each target gene across samples: **(A)** SARS-CoV-2 and Interleukins, **(B)** Chemokines, **(C)** Interferons and TNF, **(D)** The top five upregulated mitochondria-associated genes identified by RNA microarray; and **(E)** The top five downregulated mitochondria-associated genes identified by RNA microarray. Statistical significance was determined using Student’s t-test (* p<0.05, **p < 0.01, ***p < 0.001).

To further validate the stage-dependent alterations in immune response–associated genes identified in the K18-hACE2 mouse brain following SARS-CoV-2 infection, we measured absolute gene copy number in each sample through digital PCR (dPCR) analysis. We first quantified viral burden at 4 and 6 dpi by targeting the viral NP gene and found that viral RNA was not increased at 6 dpi compared with 4 dpi (**Figure 7A**), which is consistent with western blot result for SARS-CoV-2 NP protein (**Supplementary Figure S3**). These results indicate that transcriptional changes at 6dpi are not simply driven by higher viral load.

dPCR analysis of key interleukin genes, including IL-1β, IL-10, and IL-6, revealed a marked increase in transcript copy numbers at 6 dpi compared with 4 dpi, whereas IL-2 transcripts exhibited no significant change (**Figure 7B**). In contrast, chemokines involved in monocyte chemoattraction (CCL2) and neutrophil recruitment (CXCL1, CXCL2) were significantly elevated at 4 dpi but showed a substantial decline by 6 dpi. Meanwhile, CCL3 and CXCL10, both broadly recognized as interferon-responsive chemokines, exhibited a sustained and progressive increase over the course of infection. Consistently, interferon transcripts also increased in a time-dependent manner, reaching significantly higher levels at 6 dpi compared with 4 dpi (**Figure 7C**).

Together, these transcriptional patterns suggest a shift in immune state between 4 and 6 dpi, suggesting attenuation of early innate inflammatory recruitment signals, while interferon-associated and adaptive immune responses are enhanced.

Based on our GO enrichment analyses, impaired energy metabolism and mitochondrial pathways emerged as prominent features of the 6-dpi brain transcriptome. To validate these pathway-level findings, we filtered mitochondria-associated genes and selected the top five up- and down-regulated genes at 6 dpi compared with uninfected controls, ranked by adjusted p-value and log2 fold change, for dPCR analysis. Consistent with RNA microarray results, the top five upregulated mitochondrial signatures displayed time-dependent increases in transcript abundance (**Figure 7D**). Gene ontology annotation of these genes converged on processes related to mitophagy, mitochondrial fission, and mitochondrial organization, supporting progressive mitochondrial alterations associated with time-resolved infection.

Conversely, dPCR analysis of the five most downregulated mitochondrial genes revealed increased absolute transcript abundance at 6 dpi compared with uninfected controls. This apparent discrepancy, may reflect a compensatory transcriptional response to mitochondrial dysfunction, as exemplified by Ndufb7 and Ndufs6 that encode Complex I subunits, and is consistent with prior reports indicating disrupted mitochondrial integrity following SARS-CoV-2 infection (35). Despite Complex I-associated transcript increases, COXIV protein, a marker for Complex IV in the mitochondrial electron transport chain, was significantly reduced at both 4- and 6-dpi compared to the control (**Supplementary Figure S3**), suggesting complex post-infection mitochondrial remodeling, potentially including impaired assembly or stability of oxidative phosphorylation complexes at the protein level. Collectively, these data validated several key pathways significantly altered in 4- and 6-dpi, and reveal a transcriptional–translational discrepancy that may reflect compensatory host responses aimed at preserving cellular homeostasis while mounting antiviral defenses.

## 4 Discussion

In this study, we characterize the neuroinvasive potential of SARS-CoV-2 and the associated temporal transcriptional remodeling in the brains of intranasally infected a KRT18-hACE2 transgenic mice. Our findings demonstrate direct viral effects, evidenced by viral infiltration of the brain with both neurotropism and gliotropism, culminating in increased apoptosis, particularly among cortical projection neurons. In parallel, viral invasion elicits secondary immune responses, including robust inflammation marked by upregulation of chemokines involved in immune cell recruitment (e.g., CCL5, CXCL10), and activation of myeloid cells, accompanied by a shift in host defense program toward interferon-associated and adaptive immune response.

Mechanistic insight was strengthened through an integrated analytical strategy combining cytokine/chemokine profiling, RNA microarray with GO enrichment analysis, and dPCR validation of selected inflammatory and mitochondria-associated genes. This layered approach enables unbiased identification of inflammation-driven transcriptional programs and mitochondrial stress pathways, followed by absolute quantification of key immune mediators. Such analyses are particular informative in the K18-hACE2 model, given its pronounced neuroinflammatory phenotype, and allowed delineation of the temporal landscape of innate immune activation and host defense, as well as identification of potentially vulnerable molecular nodes.

Cytokine/chemokine array analysis revealed marked upregulation of immune modulators including CXCL9, CXCL10, CCL12, and IL-12p40, consistent with enhanced immune cell recruitment to infected sites. These findings are concordant with RNA microarray data at 6 dpi, which demonstrated strong activation of innate antiviral defense pathways, and align with previous reports identifying these molecules as indicator of neuroinflammation and biomarkers associated with COVID-19 disease progression (36–38). In contrast, CX3CL1 (fractalkine), a neuron-specific chemokine that mediate neuron-microglia communication and has been reported to be elevated in COVID-19 patients (39), was significantly reduced, suggesting potential disruption of protective neuron–microglia crosstalk and a maladaptive response at 6 dpi.

Noteworthily, the combined analyses revealed transcription – translation divergence across several pathways at 6 dpi, suggesting altered post-transcriptional or post-translational regulation during infection. This observation is consistent with our GO analysis showing sustained enrichment of ubiquitin-associated functions. Notably, elevated RBP4 proteins levels observed at 6dpi, whereas the retinoid signaling was among the most downregulated molecular function at 4 dpi, suggesting temporal and multi−layered modulation of retinoid-associated processes. Given prior reports implicating RBP4 in antiviral responses (34) and inflammation (40), these changes may reflect adaptive remodeling of host pathways under infectious stress. Likewise, IL-1β, TNF- α, and IL-6, cytokines frequently linked to SARS-CoV-2-associated immunopathology (6, 41, 42), exhibited time-dependent upregulations at transcript level but were not detected in cytokine profiling of 6 dpi brain tissue. As these cytokines are subjected to stringent post-transcriptional control (43, 44) and rapid turnover (45, 46), this RNA-protein discordance may indicate regulatory restraint of inflammatory signaling at later stages of infection rather than an absence of inflammatory activation. Given that chronic elevation of TNF-α and IL-6 beyond the acute inflammatory phase is known to disrupt brain homeostasis (10), impair neurogenesis (45, 47), and compromise blood–brain barrier integrity (10, 48), the observed discordance between RNA and protein levels may reflect a host regulatory mechanism that constrains excessive inflammatory signaling while maintaining antiviral defense. Importantly, such RNA–protein mismatches under severe infectious conditions highlight a regulatory layer that may shape downstream neurological outcomes. Further studies delineating this dysregulated yet restrained inflammatory signaling may contribute not only to a better understanding of COVID-19–associated neuropathology but also provide broader insight into neurodegenerative and neuroinflammatory disorders in which inflammation plays a central pathogenic role.

Temporal GO analysis further highlighted stage-specific biological alterations in host responses. At 4 dpi, transcriptional changes were primarily associated with cellular architecture, specialization, synaptogenesis, and intracellular trafficking, with enrichment of ubiquitin- and GTPase-associated functions. These enrichments, while not experimentally validated at the protein level, are consistent with prior reports indicating that viral infection can perturb vesicular trafficking and synaptic transport through modulation of protein ubiquitination (49). Given the established role of small GTPases in vesicle docking and neurotransmitter release, such alterations may contribute to disrupted neuronal communication and synaptic homeostasis (50). In parallel, enrichments of GTPase-associated pathways at this stage may also reflect virus-driven intracellular trafficking during the post-replication egress phase (51), in line with previous observations of SARS-CoV-2-associated synaptic dysfunction (52).

By 6 dpi, innate immune pathways, DNA repair and transcriptional regulatory processes dominated the transcriptional landscape, accompanied by increased numbers of TUNEL+ cells, collectively indicating heightened cellular stress and engagement of mechanisms involved in maintaining chromosome integrity and transcriptional fidelity. In contrast to early-stage alterations affecting cellular structure and synaptic function, downregulated pathways at 6 dpi were most prominently associated with energy metabolism, supported by a marked reduction in COXIV protein expression, suggesting progressive metabolic compromise and cellular exhaustion at later stages of infection. Additionally, the persistent enrichment of ubiquitin-associated pathways at this stage may reflect either continued viral exploitation of ubiquitin and UBL systems to facilitate replication, trafficking and immune evasion (53–55), or host-driven targeting of viral proteins for degradation as part of antiviral defense mechanisms (56). Notably, we also observed enrichment of ECM-associated pathways, raising the possibility that SARS-CoV-2 infection may influence ECM remodeling in brain-resident cells. Given the established relationship between ECM dysregulation and disease severity in COVID-19 lung pathology (57), analogous alterations in the brain could plausibly contribute to impaired tissue integrity and neurological manifestations, although further investigation is required.

Collectively, our findings delineate a transcriptional landscape charactered by discordant RNA-protein regulation of immune pathway-associated factors, together with evidence of metabolic and energy exhaustion at later stage of infectious, within which ubiquitin-associated pathways appears prominently engaged. The KRT18-hACE2 mouse model enables efficient and consistent viral entry to CNS and induces a robust, high penetrance neuroinflammatory and innate immune response (58, 59). Therefore, our results aim to capture the maximal transcriptional and inflammatory responses of brain tissue, rather than to model mild or resolving infection. Insights from this fulminant phenotype are particularly relevant for understanding the upper bounds of virus-driven neuroinflammation, which may be applicable to rare but clinically devastating manifestations such as severe acute neuro-COVID and COVID-associated encephalopathy, conditions that have been linked to pronounced systemic and CNS inflammatory signatures (60, 61). Importantly, since post-acute neurological sequelae can also arise following mild infection, defining pathways activated under extreme inflammatory pressure provides critical insight into mechanisms that, if incompletely resolved, may predispose to persistent neurological dysfunction. These findings establish a mechanistic framework that can be tested and refined in milder models and human systems to advance understanding of long COVID–associated neurological sequelae.

The current study has several limitations. Our layered analyses delineate pathway signatures within a global landscape of transcriptional alterations at 4 and 6 dpi; however, functional validation of the identified metabolic impairments remains limited. Accordingly, these findings should be regarded as primarily descriptive and hypothesis-generating. Further mechanistic studies incorporating cell-type–specific markers, lineage-resolved approaches, and region-specific analyses will be required to determine whether these upregulations primarily reflect viral exploitation or host-driven defense mechanism, whether they exhibit stage-dependent differences, and to delineate the respective contributions of resident and infiltrating immune cells during SARS-CoV-2–associated neuroinflammation. In addition, evaluation of sex-dependent differences in neuroinflammatory and metabolic responses may provide important biological context, particularly in light of well-documented gender discrepancy in neuroimmune regulation.

## Data Availability

The original contributions presented in the study are included in the article/**Supplementary Material**. Further inquiries can be directed to the corresponding author.
