# Supplementary material for: Neuroinflammatory and transcriptional dynamics during SARS-CoV-2 infection in KRT18-hACE2 mouse brain
Source: Front Immunol. 2026 Feb 2;17:1716597. doi: 10.3389/fimmu.2026.1716597 (PMC12907350; doi:10.3389/fimmu.2026.1716597)
Supplement: Supplementary file 1 [file DataSheet1.pdf]

Supplementary Material

Neuroinflammatory and Transcriptional Dynamics to SARS-CoV-2 Infection in KRT18-hACE2 Mouse Brain

Supplementary Figures

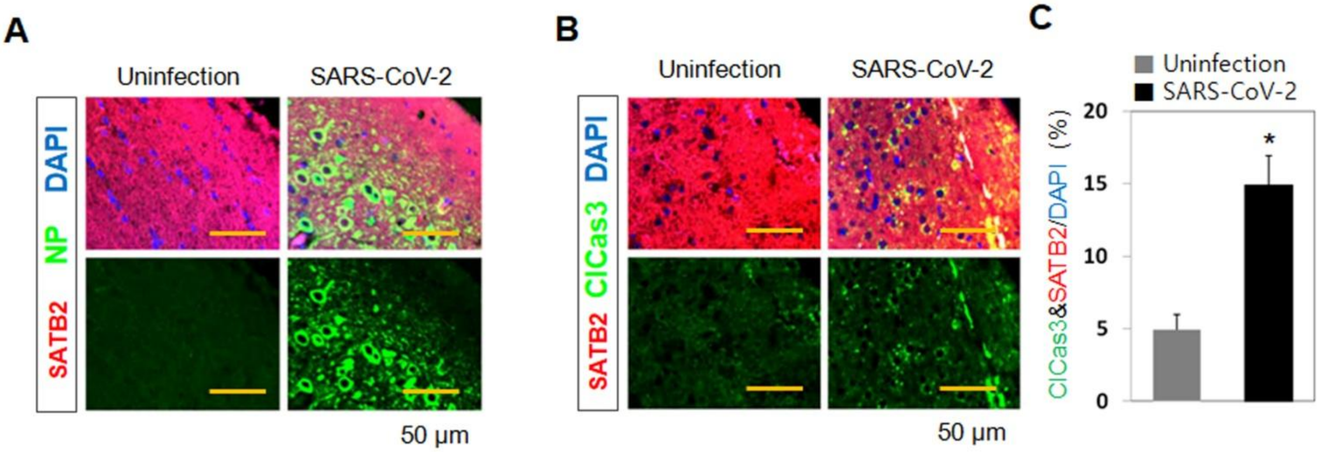

Figure S1. Neuroinvasion and Cellular Toxicity of SARS-CoV-2 in a Mouse Model

(A-B) Immunofluorescence staining of brain tissue from uninfected and infected KRT18-hACE2 transgenic mice. Each panel shows co-localization of DAPI (blue, nuclear stain) with **(A)** SARS-CoV-2 NP (green) and SATB2 (cortical neuron marker, red). **(B)** cleaved caspase-3 (ClCas3, apoptosis marker, green) and SATB2 (red). **(C)** Quantification of ClCas3-SATB2 double-positive cells in infected and infected brain. Scale bar: 50 µm. Student's t-test was used to determine statistical significance (**p < 0.01, ***p < 0.001).

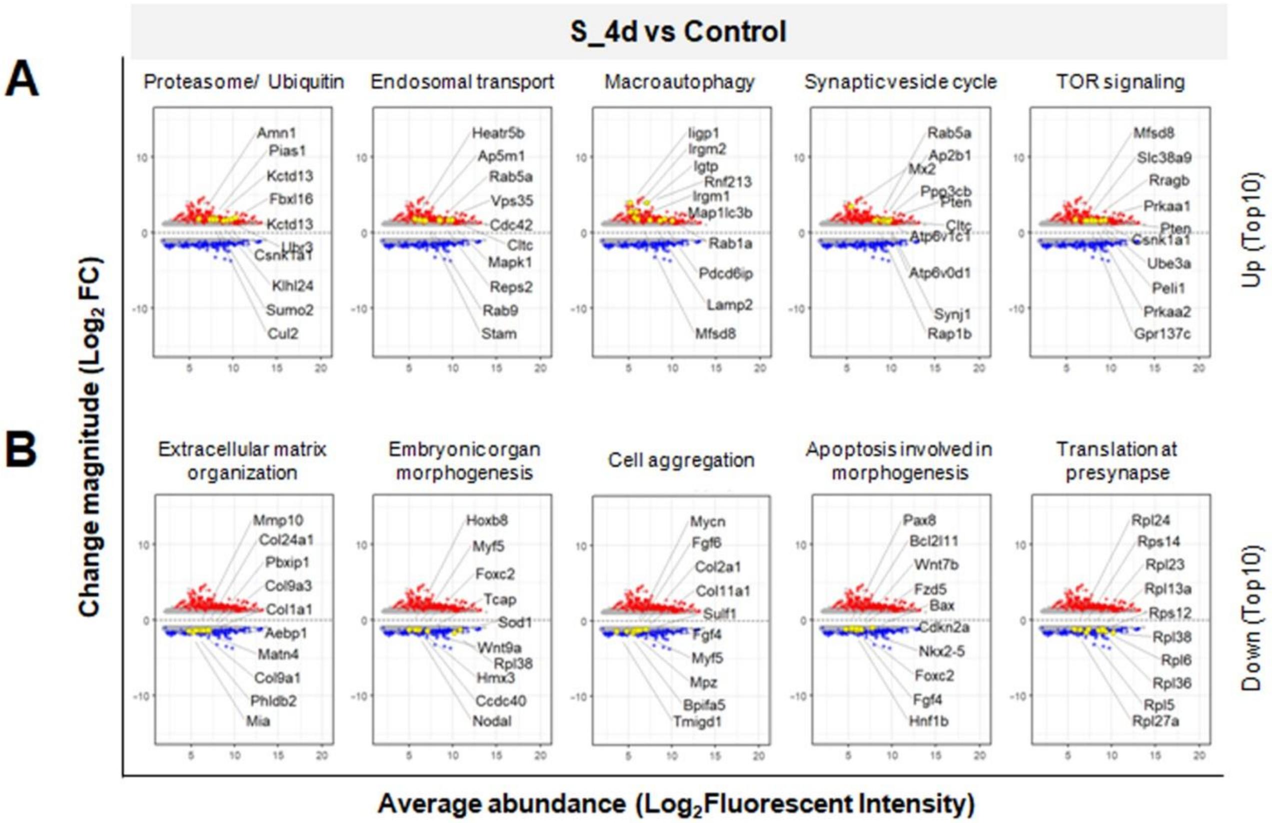

S_4d vs Control
A
Proteasome/ Ubiquitin
Endosomal transport
Macroautophagy
Synaptic vesicle cycle
TOR signaling
Up (Top10)
B
Extracellular matrix organization
Embryonic organ morphogenesis
Cell aggregation
Apoptosis involved in morphogenesis
Translation at presynapse
Down (Top10)
Change magnitude (Log2 FC)
Average abundance (Log2Fluorescent Intensity)

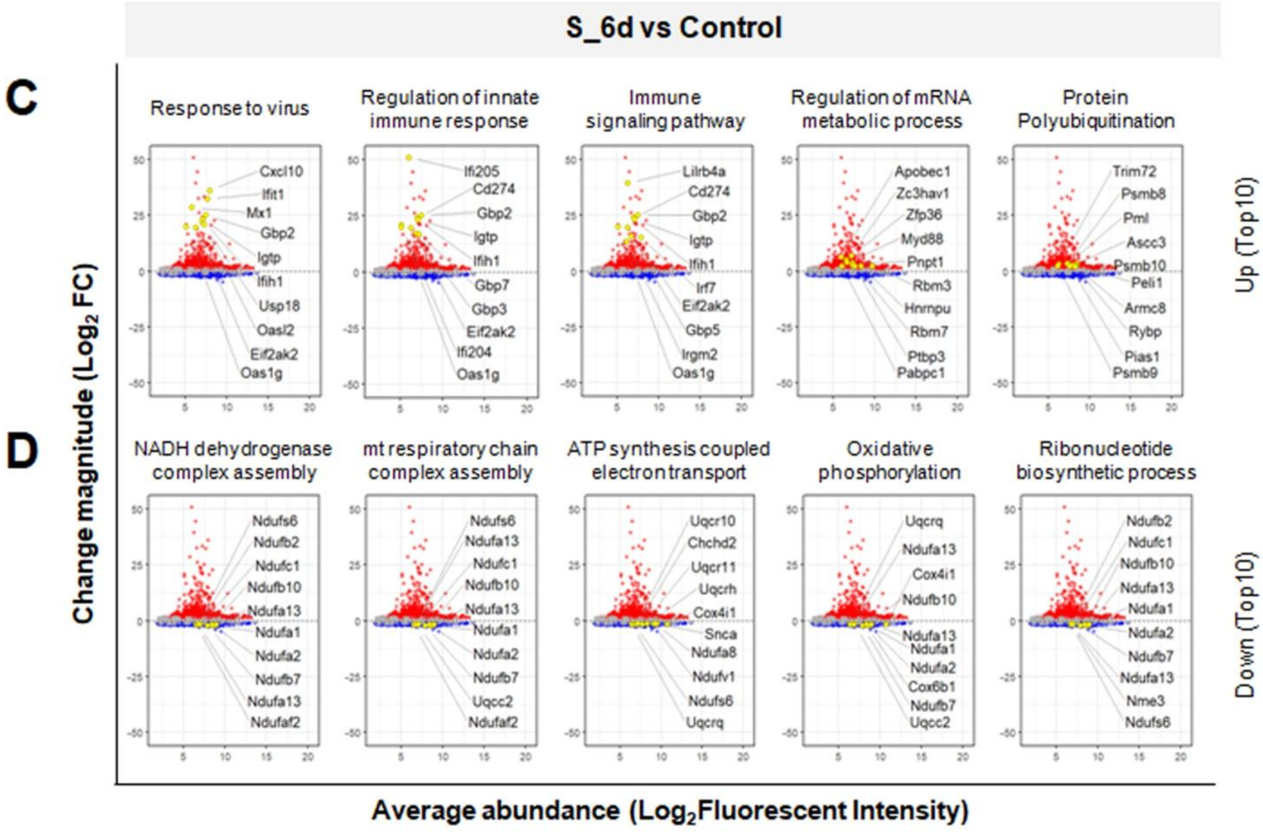

S_6d vs Control
C
Response to virus
Regulation of innate immune response
Immune signaling pathway
Regulation of mRNA metabolic process
Protein Polyubiquitination
Up (Top10)
D
NADH dehydrogenase complex assembly
mt respiratory chain complex assembly
ATP synthesis coupled electron transport
Oxidative phosphorylation
Ribonucleotide biosynthetic process
Down (Top10)
Change magnitude (Log2 FC)
Average abundance (Log2Fluorescent Intensity)

Figure S2. MA plots highlighting the top 10 gene signatures across selected biological processes. The x-axis represents average gene abundance ($\log_2$ fluorescent intensity), while the y-axis indicates the magnitude of change ($\log_2$ fold change).

(A, B) Top 10 genes **(A)** upregulated in five representative upregulated pathways, and **(B)** downregulated in five representative downregulated pathways at 4dpi compared to uninfected

(C, D) Top 10 genes **(C)** upregulated in five representative upregulated pathways, and **(D)** downregulated in five representative downregulated pathways at 6dpi compared to uninfected.

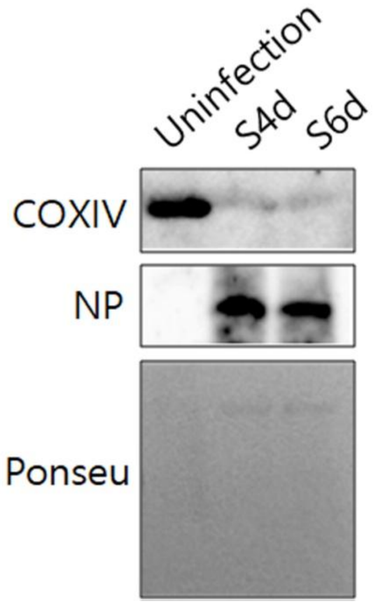

Figure S3. Disrupted mitochondrial function indicates energy exhaustion in infected brain tissue.

Western blot analysis of COXIV and SARS-CoV-2 NP in brain tissue from uninfected, 4dpi and 6dpi groups. Ponceau staining was used as a total protein loading control.

Supplementary Table

Table S1. List of primer sequences used in dPCR analysis.

**Transcript**	**Primer sequences (5'-3')**
SARS-CoV-2 N	**F:** CAATGCTGCAATCGTGCTAC<br>**R:** GTTGCGACTACGTGATGAGG
Stat3	**F:** TCCTCTATCAGCACAACCTTCG<br>**R:** ATCCGGGCAATTTCCATTGG
Igtp	**F:** TGCCTCTTCTAATCGCTCTTCC<br>**R:** AGGCCTTGCACTCTTTACTCTC
Irgm1	**F:** AGCAGAGAATAGCCAACGAGTC<br>**R:** TCGGTACACTTTCAGACACTCG
Sco1	**F:** TTGCTGCACACATGAGGTCA<br>**R:** AGCAAGCCAATCCTGCATCT
Map1lc3b	**F:** TCCACTCCCATCTCCGAAGT<br>**R:** TTGCTGTCCCGAATGTCTCC
Ndufb7	**F:** TCCGAAACCCAAGTCCAAGT<br>**R:** GATGTAGCCAGCTCCAACCT
Mrps15	**F:** AAGGACAAAGCCCACAAACG<br>**R:** TGGTCTGACGGAGGATTTTGAG

Ndufs6	**F:** AGTTCAAGCAGCACCATCAC<br>**R:** ACACCATTCACAGAGCAGTG
Ptcd3	**F:** ACTTTCCGAGATGCCCTAAGAG<br>**R:** AGCAGCACAGTCAGCAAATG
Plec	**F:** TCCTCTATCAGCACAACCTTCG<br>**R:** ATCCGGGCAATTTCCATTGG
F: Forward, R: Reverse	
